# Transcriptomic Analysis Suggests Overlapping Molecular Pathogenesis in JIA-Associated and ANA-Positive Uveitis

**DOI:** 10.3390/biom16040534

**Published:** 2026-04-03

**Authors:** Maren Kasper, Michelle Leyers, Anika Witten, Charlotte Wortmann, Regina Walet, Dirk Bauer, Melanie Schell, Kleio Petratou, Carsten Heinz, Thabo Lapp, Monika Stoll, Arnd Heiligenhaus

**Affiliations:** 1 Ophtha Lab and Department of Ophthalmology, at St. Franziskus Hospital, Hohenzollernring 74, 48145 Münster, Germanydirk.bauer@uveitis-zentrum.de (D.B.); carsten.heinz@augen-franziskus.de (C.H.);; 2 Core Facility Genomik, Universität Münster, 48149 Münster, Germanymstoll@uni-muenster.de (M.S.); 3 Department of Ophthalmology, Universität of Duisburg-Essen, 45147 Essen, Germany; 4 Eye Center, Medical Center, Faculty of Medicine, University of Freiburg, 79106 Freiburg, Germany; 5 Universität of Duisburg-Essen, 45147 Essen, Germany

**Keywords:** juvenile idiopathic arthritis, pathogenesis, transcriptome analysis, uveitis

## Abstract

Purpose: Clinical presentation of anterior uveitis affecting the iris is nearly identical in patients with juvenile idiopathic-associated uveitis (JIAU) and patients with antinuclear antibody-positive anterior uveitis (ANA-U). This study investigates whether the iris transcriptomes of patients with JIAU or ANA-U differ or reflect the clinical homogeneity. Methods: Iris biopsies were obtained from patients with JIAU (n = 31) or ANA-U (n = 9) during trabeculectomy, iridectomy, or complex cataract surgery. Frozen iris tissues were homogenized, the RNA was isolated and analyzed using a bulk RNA sequencing approach. Genes with a Benjamini–Hochberg false discovery rate (FDR) *p* ≤ 0.05 and a log_2_ fold change (FC) of >1 or <−1 were defined as significantly differentially expressed genes (DEGs). Pathway enrichment analysis was subsequently performed to characterize these DEGs. Results: The JIAU group included nine male and 22 female patients (median age 10.2 [IQR 7.2, 13.7] years), while the ANA-U group consisted of one male and eight female patients (median age 10.2 years (IQR [6.1, 11.7]) years). No DEGs were identified when comparing the iris transcriptomes of JIAU and ANA-U directly. Subgroup analyses based on disease activity revealed 35 DEGs in ANA-U, one DEG in JIAU. Inter-group comparisons based on uveitis activity status yielded no DEGs. In the merged JIAU/ANA-U cohort, 28 DEGs were upregulated in the active vs. inactive uveitis. Regarding secondary glaucoma, 12 DEGs were identified in JIAU, and 15 DEGs were identified in the corresponding ANA-U subgroup. Inter-group comparisons based on glaucoma status yielded no DEGs. In the merged JIAU/ANA-U cohort, 20 DEGs were identified between patients with and without glaucoma. Conclusions: Our findings reveal only marginal differences in gene expression patterns in iris tissues from JIAU and ANA-U patients. These results suggest a high degree of similarity in the underlying inflammatory processes of both uveitis subsets.

## 1. Introduction

Uveitis remains a leading cause of preventable blindness in both children and adults. In the pediatric population, idiopathic anterior uveitis (IAU) accounts for approximately 30–40% of all cases [1]. Parallel to this, juvenile idiopathic arthritis (JIA)—the most prevalent pediatric rheumatic disease—is complicated by associated uveitis (JIAU) in up to one-fifth of patients [2].

JIAU is typically characterized by the presence of antinuclear antibodies (ANAs), an insidious onset, and a chronic, relapsing course that frequently leads to vision-threatening complications [3,4]. While established clinical guidelines emphasize the necessity of multidisciplinary care between ophthalmologists and rheumatologists [5,6,7], long-term prognosis is often hindered by a poorly understood pathogenesis involving a complex interplay of innate and adaptive immune responses [8,9].

As the ANA status per se is neither a specific cause nor a clinical manifestation of uveitis, a routine ANA determination is not recommended, except in cases with signs of systemic diseases such as JIA [10]. Thus, ANA determination is not usually done in clinical monitoring of IAU patients.

Intriguingly, JIAU and ANA-positive IAU are phenotypically indistinguishable; they share near-identical clinical courses, complication rates (e.g., secondary glaucoma, cataract, band keratopathy), and therapeutic responses to corticosteroids and disease-modifying antirheumatic drugs (DMARDs). Consequently, these two entities are managed with identical treatment protocols [11,12,13].

Genetic studies further support a shared etiology, as chronic anterior uveitis without arthritis shares key HLA alleles with JIAU, suggesting that IAU may simply be an early manifestation of JIAU [14]. Furthermore, analyses of aqueous humor, tears, and iris cellular infiltrates have yielded largely overlapping cytokine profiles for both groups [15,16,17].

Despite these clinical and genetic parallels, it remains unknown whether these conditions share a common molecular fingerprint at the tissue level.

Anterior uveitis affects the iris and ciliary body. In previous immunohistochemical studies, a cellular infiltrate of predominantly B cells in iris biopsies of JIA and ANA-U patients could be shown [17,18,19,20,21]. Presence of B-cells in the iris tissue of JIAU patients was confirmed in a bulk RNA seq study [22]. To elucidate the intraocular proinflammatory landscape and determine the degree of molecular divergence between JIAU and ANA-positive IAU (ANA-U), we performed comprehensive bulk RNA sequencing to characterize the global iris transcriptome.

## 2. Materials and Methods

### 2.1. Patients and Sample Collection

All study participants were diagnosed with chronic anterior uveitis characterized by an insidious onset of flare associated with juvenile idiopathic arthritis (JIA), and patients with ANA-positive anterior uveitis but without arthritis were included in the study. Exclusion criteria included other uveitis entities (e.g., infectious), other anatomic uveitis subtypes, or the presence of other associated systemic immune-mediated diseases. JIA was diagnosed by collaborating pediatric rheumatologists in accordance with the International League of Associations for Rheumatology (ILAR) criteria [23], and uveitis (including activity) and related complications (e.g., glaucoma) were assessed in accordance with the recommendations of the Standardization of Uveitis Nomenclature (SUN) Working Group [24].

Iris biopsy specimens were collected during elective trabeculectomy, iridectomy, or complex cataract surgery under general anesthesia. Prior to surgery, prednisolone acetate 1% eye drops were given 5 times daily for one week. Iris specimens were immediately snap-frozen in liquid nitrogen and stored at −80 °C until further processing. Samples were collected at the Department of Ophthalmology at St. Franziskus Hospital, Münster. This study was conducted in accordance with the principles of the Declaration of Helsinki and was approved by the local ethics committee (protocol code 2020-614-f-S, 2015-013-f-S). Written informed consent was obtained from patients and parents prior to surgery.

### 2.2. RNA Preparation, Library Construction and Sequencing

Frozen iris tissue samples were homogenized mechanically using a pestle. RNA isolation was followed by using the Direct-zolTM RNA MicroPrep kit. (Zymo Research Group GmbH, Freiburg, Germany), A Bioanalyzer or TapeStation (Agilent, Santa Clara, CA, USA) was used to determine the RNA Integrity Number (RIN). Total RNA libraries were constructed after rRNA removal using the NEBNext^®^ rRNA Depletion Kit and the NEBNext^®^ Ultra II Directional RNA Library Prep Kit (both from New England Biolabs GmbH, Frankfurt am Main, Germany), following the manufacturer’s instructions. Paired-end sequencing was performed on the Illumina NextSeq2000 platform at a depth of 40 million pairs per sample.

### 2.3. Bioinformatic Analysis

Raw sequencing reads were introduced into the RNA bioinformatics pipeline. Quality control was performed on the raw FASTQ files using FastQC 0.12.1 [25] and MultiQC 1.30 [26]. FASTP 0.24.0 was used to quality filter reads and trim adapters [27] using the –g PolyG tail-removing option to remove overrepresented poly-G tails. For alignment, we used the GRCh38.p14 v113 reference genome from the Ensembl-database [28], as well as the matching GTF files for annotation purposes (also from the Ensembl database). The read alignment itself was performed using the splicing-aware STAR aligner 2.7.11b [29], which sorted the output reads by coordinates during the alignment process. The quality of alignment was assessed via a combination of different tools: IGV 2.17.3 for Linux [30,31] for random inspection of the samples, RSeQC 5.0.1 [32,33], Samtools 11.19.2 [33] and the STAR alignment statistics for each sample. Due to high duplicate rates after alignment, MarkDuplicates in Picard 3.1.1 (Picard Toolkit, Broad Institute, GitHub Repository, http://broadinstitute.github.io/picard/, 2019, accessed on 16 January 2025) function was applied to remove duplicates for downstream analysis. Each aligned sample was checked with the dupRadar 1.38.0 R package [34] before applying this function. FeatureCounts (subread 2.0.6) [35] was used to count the reads mapped to the reference genome. To address multimapping issues, we filtered secondary alignments with Samtools before running FeatureCounts again.

### 2.4. Identification of Differentially Expressed Genes

Gene count matrices from FeatureCount were used further for differential gene expression (DEG) analysis in R with the DESeq2 1.48.0 Bioconductor package [36]. Confounding factors, such as the type of surgery the patients underwent (especially trabeculectomy) or the sample processing batch, were taken-into-account for the linear model of the DESeqDataSet object (~surgery + batch + uveitis-subgroup). Genes with low or zero counts (fewer than ten counts in at least 18 samples) were removed before running DESeq2.

Principal component analysis (PCA) was used to assess data quality and check for additional biases and confounding factors. For visualization, the data were transformed using the variance-stabilizing transformation function. Genes were considered significant if they had a Benjamini–Hochberg adjusted *p*-value (FDR) of less than 0.05 and a log_2_ fold change (FC) greater than 1 or less than −1. Hierarchical cluster analysis was performed on significant DEGs.

### 2.5. Pathway Enrichment Analysis

The g:GOSt tool from g:Profiler (gprofiler2 v0.2.3) [37,38] was used to identify significantly enriched Gene Ontology (GO) terms and pathways. For analysis, we incorporated the gprofiler2 R package version 0.2.3 [39] into our pipeline. The package performs statistical enrichment analysis to identify overrepresented GO terms and biological pathways in user-provided gene lists using cumulative hypergeometric tests [40,41]. We created two lists containing significantly up- and downregulated genes as input for pathway enrichment analysis with the gprofiler2 package. We applied a threshold of 0.05 for determining significant terms, along with the FDR correction method. The list of all genes analyzed with DESeq2 was used as a custom background, excluding all genes with an “NA” entry for the *p*-value in the results table. Only manually reviewed GO annotations were considered. The output of gprofiler2 was fine-tuned by removing all pathway terms with a term size less than 5 or greater than 350 to exclude pathways that are too small or too large with limited interpretative value [42]. To enhance interpretability and reduce background noise, only functional categories containing at least three genes from the input set were included in the analysis. The sources included in the analysis were GO Molecular Function, GO Biological Process, and GO Cellular Component.

## 3. Results

### 3.1. Clinical Data

Clinical demographics for the 40 pediatric patients included in this study are summarized in Supplementary Table S1. The cohort comprised patients with JIAU (n = 31) and ANA-U (n = 9). At the time of surgery, the median age was 10.2 years (IQR [7.2, 13.9]) for the JIAU group and 10.2 years (IQR [6.1, 11.7]) for the ANA-U group. Uveitis duration was longer in the JIAU group (median 5.6 years; IQR [3.1, 9.6]) compared to the ANA-U group (median 2.1 years; IQR [1.5, 5.5]). Both groups were predominantly female and shared a similar immunological profile: ANA-positive, RF-negative, and HLA-B27-negative. Within the JIAU group, all patients were diagnosed with oligoarticular JIA, categorized as either persistent (n = 21) or extended (n = 10) subtypes.

### 3.2. Clinical Status and Complications

While a minimum of three months of clinical inactivity (<0.5+ cells per SUN criteria) was the standard prerequisite for surgery, a subset of patients (n = 5 JIAU; n = 4 ANA-U) required urgent intervention. Indications for urgent surgery included rapidly progressing mature cataracts, anterior chamber flattening, or refractory glaucoma with characteristic optic disk changes despite maximal medical therapy.

**Figure 1:**
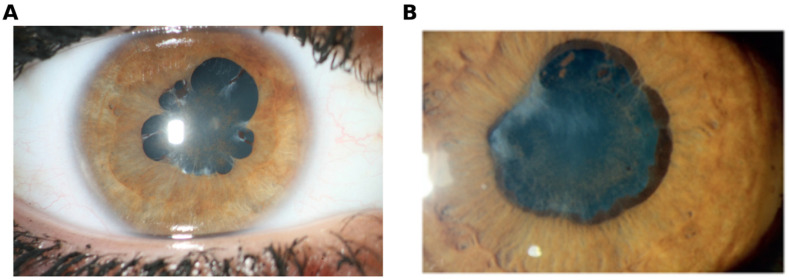
Representative slit-lamp appearance of (**A**) juvenile idiopathic arthritis (JIA)-associated anterior uveitis versus (**B**) ANA-positive anterior uveitis. For both, there are several posterior synechiae and a fibrovascular membrane on the lens.

All participants presented with at least one uveitis-related complication, such as posterior synechiae or band keratopathy (Figure 1). Most patients developed cataracts (JIAU: n = 30; ANA-U: n = 8), secondary glaucoma (JIAU: n = 19; ANA-U: n = 5), posterior synechia (JIAU: n = 25; ANA-U: n = 8), or band keratopathy (JIAU: n = 22; ANA-U: n = 4). At the time of sampling, most patients were on systemic immunomodulatory therapy, including conventional synthetic (cs) and/or biological (b) disease-modifying antirheumatic drugs (DMARDs) (Supplementary Table S1).

### 3.3. Differential Iris Transcriptome of Patients with JIAU Versus ANA-U

**Figure 2:**
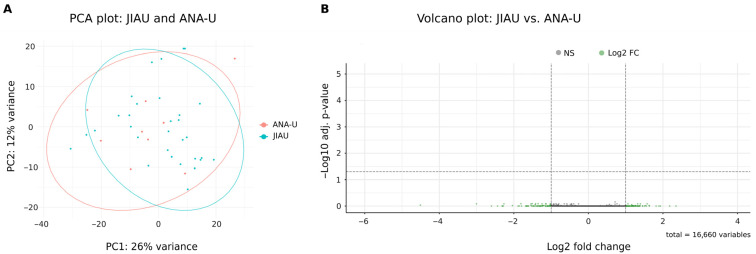
Iris transcriptome of JIAU versus ANA-U patients. (**A**) Principal component analysis (PCA) was performed on normalized gene expression data to evaluate variability among samples and identify clustering patterns. Each point represents an individual patient. JIAU (n = 31) and ANA-U (n = 9). (**B**) The volcano plot displays the distribution of gene expression changes identified by comparing the JIAU vs. ANA-U groups, illustrating transcriptional differences associated with uveitis. DEGs (red dots) are defined as an adjusted *p*-value of *p* ≤ 0.05, and a log_2_FC of >1 (upregulated) or log_2_FC < −1 (downregulated). Genes with log_2_FC > 1 or <−1 but *p* > 0.05 (not significant) are shown as green dots. Genes that do not pass these thresholds are shown in gray.

Principal component analysis (PCA) of the iris transcriptomes revealed that JIAU and ANA-U samples exhibited overlapping profiles without distinct clustering (Figure 2A). In agreement with these findings, no significant DEGs were identified between the two groups (Figure 2B; Supplementary Table S2).

### 3.4. Differential Iris Transcriptome of Patients with JIAU Versus ANA-U During Active or Inactive Uveitis

**Figure 3:**
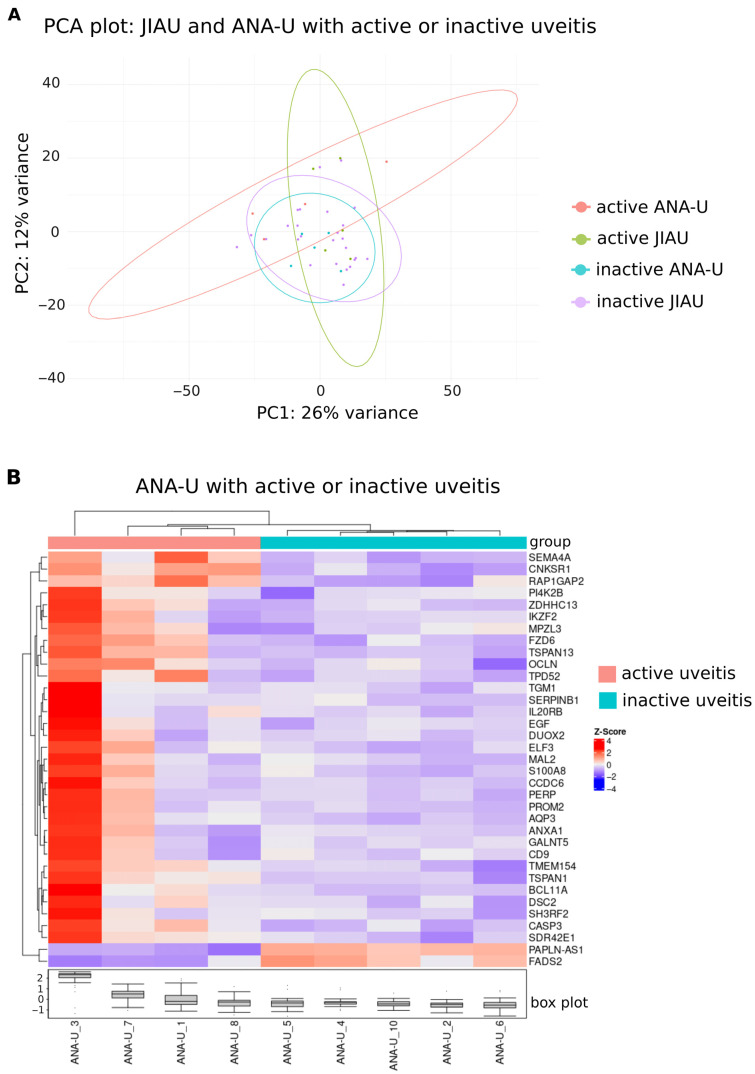
Iris transcriptome of JIAU and ANA-U patients during active and inactive uveitis. (**A**) PCA plot of iris samples, color-coded by clinical group (JIAU vs. ANA-U) and disease activity status (active vs. inactive). (**B**) Hierarchical clustering heatmap visualizing 35 genes in the ANA-U group, which were significantly differentially expressed (upregulated n = 33; downregulated n = 2) when comparing active uveitis (n = 4) and inactive uveitis (n = 5) samples. Each row represents one gene, and each column represents one sample. The z-score represents a gene’s expression in relation to its mean expression by standard deviation units (red: upregulation and blue: downregulation).

When categorized by clinical activity (defined as <0.5+ anterior chamber cells), PCA of JIAU and ANA-U samples showed overlapping distributions, with low variance (Figure 3A). However, active ANA-U samples exhibited a trend toward separation from the inactive ANA-U cluster. Intra-group analysis of JIAU patients revealed significant upregulation of *DENND11* during active uveitis (Supplementary Table S2). Inter-group comparison of active cases identified a significant upregulation of *SEMA4A* in the JIAU group compared to the ANA-U group. In contrast, no significant DEGs were identified between the groups during periods of clinical inactivity (Supplementary Table S2).

Hierarchical clustering analysis of the ANA-U group revealed two distinct branches corresponding to uveitis activity (Figure 3B). Comparison between active and inactive states identified 35 DEGs: 33 were significantly upregulated (including *S100A8* and *IL20RB*) and two were significantly downregulated (*FADS2* and *PAPLN-AS1*) during the active phase (Figure 3B, Supplementary Figure S1, Supplementary Table S2). Gene Ontology analysis indicated that these DEGs are primarily involved in cell–cell junction processes and calcium ion binding (Supplementary Table S4).

### 3.5. Differential Iris Transcriptome of Patients Within the Merged JIAU/ANA-U Group During Active or Inactive Uveitis

**Figure 4:**
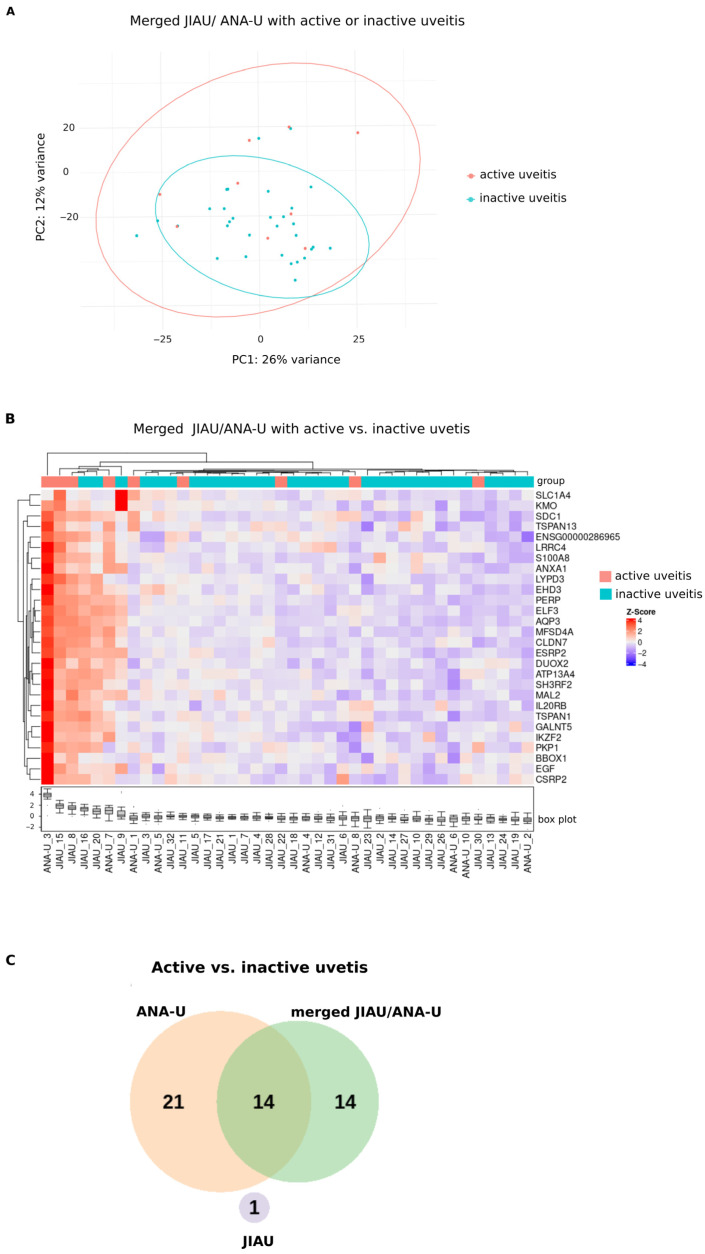
Iris transcriptome of the merged JIAU/ANA-U samples with active versus inactive uveitis, and overlapping genes with JIAU and ANA-U samples during active uveitis. (**A**) PCA plot showing merged JIAU/ANA-U samples marked by different colors depending on uveitis activity status (active or inactive) at the time of sample collection. (**B**) Hierarchical clustering heatmap visualizing 28 genes in the merged JIAU/ANA-U group, which were significantly upregulated when comparing active uveitis (n = 9) and inactive uveitis (n = 31) samples (Supplementary Table S2 and Supplementary Figure S1). Each row represents one gene, each column represents one sample. The z-score represents a gene’s expression in relation to its mean expression by standard deviation units (red: upregulation and blue: downregulation). (**C**) The Venn diagram depicts the number of unique and shared DEGs identified across the compared groups. Each circle represents one group: ANA-U, JIAU, or JIAU/ANA-U with active versus (vs.) inactive uveitis. The overlapping regions of the circles indicate the same genes that are regulated within the groups (see Supplementary Table S3).

Given the transcriptomic similarity between the iris samples of the JIAU and ANA-U groups, the cohorts were merged into a single JIAU/ANA-U group to increase statistical power. Differential gene expression was subsequently re-evaluated in relation to uveitis activity (Figure 4; Supplementary Table S2). PCA showed that samples from both active and inactive subgroups were interspersed without clear separation (Figure 4A). However, three samples of patients with active uveitis (ANA-U-3, JIAU-15, and JIAU-8) exhibited a cluster in the hierarchical clustering heatmap (Figure 4B). Differential expression analysis identified 28 genes significantly upregulated during active uveitis in the merged cohort (Figure 4B, Supplementary Figure S1, Supplementary Table S2). Gene Ontology analysis indicated that these DEGs are primarily involved in cell–cell junction processes (Supplementary Table S4).

Among the DEGs identified in the merged JIAU/ANA-U group (e.g., *S100A8*, *IL20RB*, and *MAL2*), 14 overlapped with those found in the ANA-U group (e.g., *S100A8*, *ANXA1*, and *IKZF2*). ANA-U patients uniquely expressed 21 DEGs (e.g., *SEMA4A* and *CD9*). In contrast, the JIAU group shared no common DEGs with the other groups and uniquely expressed *DENND11* (Figure 4C, Supplementary Table S3).

The upregulated genes identified in patients with active uveitis include both proinflammatory functions—such as the alarmin *S100A8* promoting granulocytes/monocyte infiltration [43] and *SDC1* involved in leukocyte endothelial adhesion [44], as well as anti-inflammatory potential via, e.g., *ANXA1* [45,46,47], *CD9* [46], and *IKZF2* [48].

### 3.6. Differential Iris Transcriptome of Patients with JIAU Versus ANA-U with or Without Secondary Glaucoma

**Figure 5:**
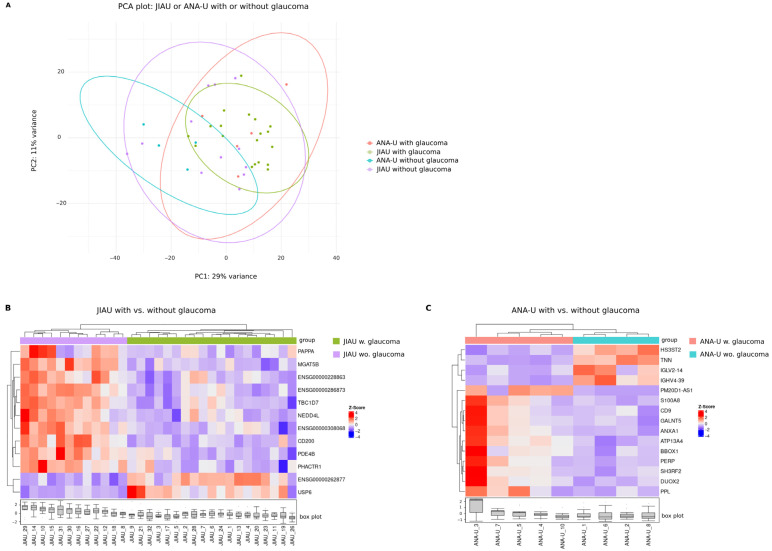
Iris transcriptome of JIAU and ANA-U patients with versus without glaucoma. (**A**) PCA plot showing the samples of the JIAU and ANA-U groups marked by different colors depending on the presence of glaucoma at the time of sample collection. Hierarchical clustering heatmap visualizing differentially expressed genes in the (**B**) JIAU and (**C**) ANA-U groups, comparing samples with (w) glaucoma (JIAU n = 19 and ANA-U n = 5) versus without (wo) glaucoma (JIAU n = 12 and ANA-U n = 4). In the glaucoma versus non-glaucoma comparison, 12 (JIAU) and 11 (ANA-U) genes were significantly upregulated, while 10 (JIAU) and 4 (ANA-U) genes were significantly downregulated (Supplementary Table S2 and Supplementary Figure S2). Each row represents one gene, each column represents one sample. The z-score represents a gene’s expression in relation to its mean expression by standard deviation units (red: upregulation and blue: downregulation).

Secondary glaucoma is a major vision-threatening complication in patients with JIAU and ANA-U. To investigate its transcriptomic signature, we performed comparative analyses both between and within these two groups, stratified by the presence of glaucoma. In the PCA plot, samples from both the JIAU and ANA-U cohorts generally appeared interspersed regardless of glaucoma status (Figure 5A). However, ANA-U samples with and without glaucoma formed clusters separately. No DEGs were identified when comparing JIAU and ANA-U patients with glaucoma, nor when comparing the two groups without glaucoma (Supplementary Table S2). In contrast, hierarchical clustering revealed that samples within both the JIAU and ANA-U groups branched distinctly according to the presence of glaucoma (Figure 5B,C). In JIAU patients, we identified 12 DEGs associated with glaucoma: two were significantly upregulated (including *USP6* and a novel long non-coding RNA transcript), and ten were downregulated (e.g., *PAPPA* and *CD200*) compared to those without glaucoma (Figure 5B, Supplementary Figure S2, Supplementary Table S2). Within the ANA-U group, we identified 15 DEGs associated with glaucoma: 11 genes were significantly upregulated, and four were downregulated (e.g., *IGHV4-39* and *TNN*) in patients with glaucoma relative to those without glaucoma (Figure 5C, Supplementary Figure S2, Supplementary Table S2).

### 3.7. Differential Iris Transcriptome of Patients Within the Merged JIAU/ANA-U Group with or Without Glaucoma

When evaluating the merged JIAU/ANA-U cohort by glaucoma status, samples exhibited moderate separation in the PCA plot (Figure 5A). Hierarchical clustering further distinguished the groups, with samples—excluding JIAU-8—partitioning into two distinct branches based on the presence of glaucoma (Figure 5B). Within the JIAU/ANA-U group, we identified 20 DEGs associated with glaucoma: four genes were significantly upregulated (e.g., *CCN2* and *GJA3*) and 16 were downregulated (including several immunoglobulin variable *IG_V* genes, *THEMIS2*, *CIITA*, and *NEDD4L*) in the glaucoma subgroup (Supplementary Figure S2 and Supplementary Table S2). Pathway enrichment analysis did not yield significant results, as no biological categories met the minimum threshold of three matching genes.

**Figure 6:**
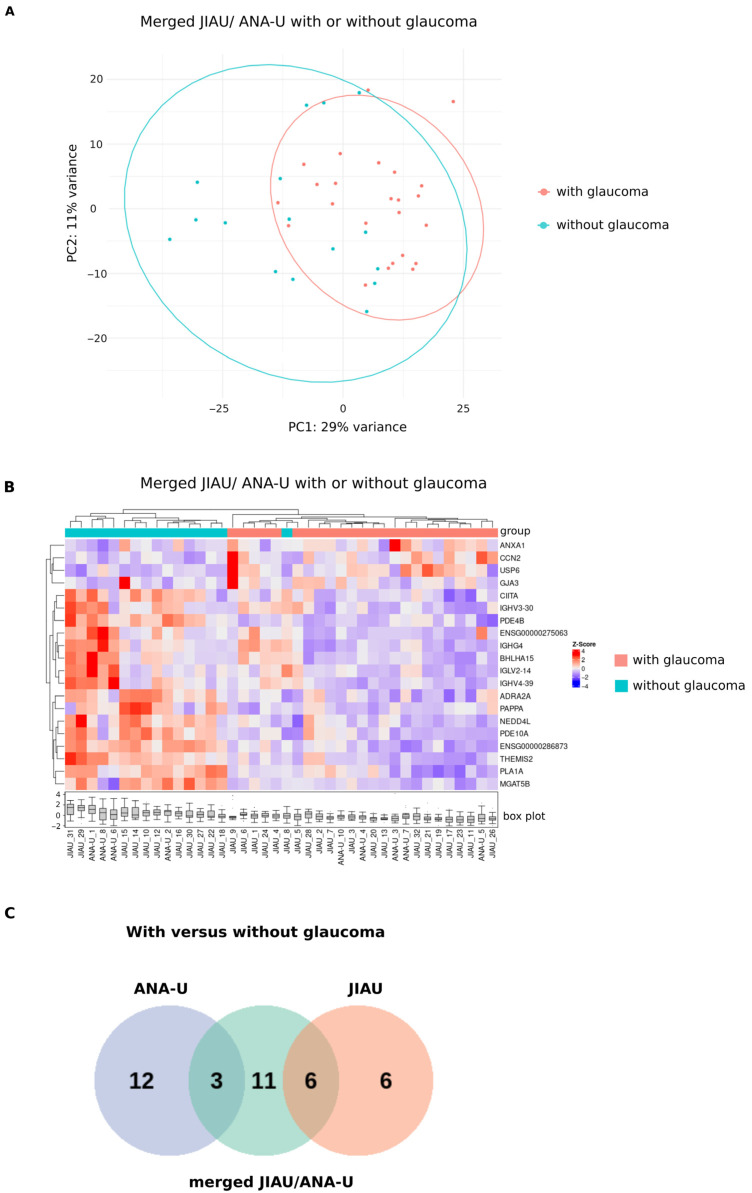
Iris transcriptome of merged JIAU/ANA-U patients with glaucoma versus without glaucoma. (**A**) PCA plot showing merged JIAU/ANA-U samples marked by different colors depending on glaucoma status [with (w) or without (wo)] at the time of sample collection. (**B**) Hierarchical clustering heatmap visualizing 4 genes in the merged JIAU/ANA-U group, which were significantly upregulated when comparing w glaucoma (n = 24) and wo glaucoma (n = 16) samples (Supplementary Table S2 and Supplementary Figure S2). Each row represents one gene, each column represents one sample. The z-score represents a gene’s expression in relation to its mean expression by standard deviation units (red: upregulation, blue: downregulation). (**C**) The Venn diagram depicts the number of unique and shared DEGs identified across the compared groups. Each circle represents one group: ANA-U, JIAU, or JIAU/ANA-U w glaucoma versus (vs.) wo glaucoma. The overlapping regions of the circles indicate the same genes that are regulated within the groups (see Supplementary Table S5).

Among the DEGs identified in the merged JIAU/ANA-U group, three genes (*ANXA1*, *IGHV4-39*, and *IGLV2-14*) were shared with the ANA-U group, and six genes (e.g., *USP6*, *PAPPA*, and *NEDD4L*) overlapped with the JIAU group. Furthermore, 12 DEGs were uniquely identified in samples of the ANA-U group (e.g., *S100A8*), 11 in the merged JIAU/ANA-U group (e.g., *CCN2*), and six in the JIAU group (e.g., *CD200*) (Figure 6 and Supplementary Table S5).

Similar to uveitis activity, the upregulated genes identified in patients with glaucoma include both proinflammatory (e.g., *S100A8* [43] and *CCN2* [49]) and anti-inflammatory functions (*ANXA1* [45,46,47] and *CD9* [45,46,47]). Furthermore, DEGs such as *PAPPA* and *TNN*, relevant for extracellular matrix (ECM) remodeling [50], are downregulated; the disturbance of ECM remodeling, particularly in trabecular meshwork cells, is a key process in glaucoma pathogenesis [51]. In addition, markers of adaptive immunity, e.g., *CIITA* required for MHC II restricted antigen presentation [52] and *IGHG4* pointing to a local presence of plasma cells [22], are downregulated.

## 4. Discussion

In the current study, a comparison of the iris transcriptomes of ANA-U and JIAU patients revealed no significant differences when samples were analyzed without stratification for uveitis activity, ocular complications, or therapeutic regimens. This finding reinforces clinical observations of equivalence between these two entities [11], suggesting that the clinical course and treatment responses in JIAU and ANA-U are largely comparable. While several studies have previously characterized the peripheral [53] or local cytokine milieu in the aqueous humor [8,16,22,54] and tears [15,55,56], these proteomic approaches often varied in methodology, clinically heterogeneous cohorts and statistical approaches, which may substantially hamper direct comparison of their findings. Furthermore, although previous transcriptomic studies of ocular tissues in JIA patients have provided insights into local immunological processes [22,57], they did not directly compare ANA-U and JIAU within a single cohort, nor did they focus specifically on the iris—the tissue primarily involved in the disease.

Subgroup analysis in the present study revealed elevated expression of *DENND11* and *SEMA4A* in JIAU patients with active uveitis compared to their ANA-U counterparts. While *DENND11* is ubiquitously expressed, a role in neuritogenesis after ischemia has been described [58]. Studies on immunomodulatory function are lacking and do not allow any connection to uveitis activity to be made.

The semaphorin family contains critical immunoregulators. *SEMA4A* is known to facilitate T-cell co-stimulation [59], while *SEMA3A* has demonstrated immunomodulatory capacity in experimental autoimmune uveitis (EAU) [60,61] and other autoimmune disorders [62]. Notably, in axial spondyloarthritis, peripheral *SEMA3A* levels correlate with systemic disease activity and the presence of uveitis [63]. During inactive uveitis, no transcriptomic differences were detected between the two entities, suggesting a convergence of transcriptional profiles during clinical remission.

Interestingly, comparing active versus inactive states yielded significantly more DEGs within the ANA-U group than within the JIAU group. This discrepancy may be attributed to the significantly higher proportion of JIAU patients receiving DMARDs, as both uveitis and arthritis show higher baseline disease activity and severity.

This discrepancy may be attributable to the significantly higher proportion of JIAU patients receiving DMARDs, as both uveitis and arthritis show higher baseline activity and disease severity.

An alternative interpretation is that irreversible damage to iris tissue in JIAU patients remains the upregulation of inflammation- and damage-associated genes even during inactive disease [22]. In a ceiling effect, additional disease activity produces only a limited further change in the expression signal. Similar observations were made in RNA-seq studies with synovial tissue of patients with rheumatoid arthritis [64,65].

In addition, Triaille et al. showed that various DMARDs consistently downregulate immune cell activation genes in RA synovium, with stronger effects in treatment responders. Thereby, DMARD therapy leads to a dampening of transcriptomic activity and prevents additional expression increases with recurrent disease activity [66].

The DEGs identified in active ANA-U patients are associated with the modulation of cell–cell junctions, likely reflecting tight junction dysfunction and subsequent breakdown of the blood-ocular barrier [64,65,67]. Additionally, we observed an enrichment of calcium-ion binding processes, particularly involving *S100A8* during active uveitis. *S100A8* and its heterodimer *S100A9* are primarily expressed in myeloid cells, such as macrophages and granulocytes, and play an important role in inflammatory processes [68]. The presence of S100-A8 positive granulocytes and monocytes in ocular tissue was shown in experimental autoimmune uveitis (EAU) [69], and in particular in the iris–ciliary body in an experimental model of LPS-induced uveitis (EIU) [43].

This aligns with evidence positioning S100A8/A9 proteins as potent proinflammatory biomarkers in multiple sclerosis, rheumatoid arthritis, JIA, and uveitis, reflecting the recruitment of monocytes to the local and peripheral immune response [16,56,70,71]. Beyond *S100A8*, the elevated expression of *SERPINB1*, *ANXA1*, *FADS2*, and *DUOX2* further implicates monocyte involvement. T-cell activity was suggested by increased expression of *SEMA4A*, *CD9*, and *IKZF2*. Notably, IKZF2 (Helios) is a marker for regulatory T-cells (Tregs) [72], potentially indicating a local counter-regulatory mechanism against inflammation. Furthermore, the presence of B-cells was indicated by the expression of *BCL11A*, *TSPAN1*, *TSPAN13*, *GALNT5*, *IL20RB*, and *PROM2*, corroborating earlier findings [22,57].

To increase statistical power, JIAU and ANA-U data were merged for a combined analysis of uveitis activity. This re-analysis reaffirmed the involvement of monocytes, T-cells, and B-cells, alongside alterations in tight junctions—evidenced by the upregulation of *PKP1*, *SDC1*, and *CLDN7*, which are known to modulate immune cell recruitment and barrier integrity.

Secondary glaucoma is a major vision-threatening complication associated with chronic uveitis. While no DEGs were found when comparing the two uveitis subgroups directly in the context of glaucoma, intra-group analyses identified several DEGs associated with local inflammation. Although these were insufficient for robust pathway enrichment, we observed reduced *TNN* (Tenascin-N) expression in ANA-U patients with glaucoma. Tenascin-C, another member of this extracellular matrix (ECM) family, plays a known role in experimental autoimmune glaucoma [73,74] and is elevated in human glaucomatous eyes [75,76].

While glaucoma is typically a consequence of chronic inflammation, it often manifests after clinical inactivity is achieved [77,78,79]. In line with this notion, our analysis of the merged JIAU/ANA-U group suggested reduced pro-inflammatory activity in patients with secondary glaucoma. However, there is a sampling bias to mention: the non-glaucoma group contained a higher proportion of samples with active uveitis at the time point of surgery (n = 6) compared to the glaucoma group (n = 3).

The results of the comprehensive bulk RNAseq analysis confirm the previous clinical observations outlining a similar clinical course of uveitis in the two patient groups, and also response to treatment. The results are relevant for clinical care, particularly diagnostic measures and treatment modalities in the future.

## 5. Limitations of Our Study

Several limitations warrant consideration. First, unequal distributions of gender and age between cohorts may serve as confounding factors. Second, the modest and unequal sizes of the JIAU and ANA-U groups may have limited the statistical power to detect subtle differential expression.

Third, the reduced library complexity due to PCR duplicates may have hindered the detection of lower expressed transcripts.

Furthermore, the lack of independent validation (e.g., via RT-qPCR) limits the generalizability of these transcriptomic findings, but it was not feasible due to a lack of further samples. A comparison with healthy iris tissue would be desirable, but for ethical reasons, it is not possible to obtain healthy human iris tissue.

Finally, the influence of anti-inflammatory therapy cannot be fully excluded; nearly all patients received topical corticosteroids preoperatively, and the majority were on systemic DMARDs, in particular MTX and TNF-alpha inhibitors. The significant differences in therapeutic regimen between the JIAU and ANA-U cohorts likely influenced the iris transcriptome and increased overall heterogeneity. Additionally, the presence of other uveitis-related eye complications may have increased transcriptome heterogeneity.

As the bulk RNA Sequencing approach used in the current study does not resolve transcriptional profiles at the single-cell level, gene expression patterns attributed to specific immune cell types should not be overinterpreted. Future studies should use single-cell RNA sequencing methods to overcome this limitation.

## 6. Conclusions

In conclusion, our results demonstrate that JIA-associated and ANA-positive uveitis share a convergent transcriptional signature. This molecular similarity likely reflects a common final inflammatory pathway, providing a biological basis for the observed parallels in their clinical progression and their equivalent responses to DMARD therapy.

## Data Availability

The data presented in this study are available on request from the corresponding authors.

## Supplementary Materials

The following supporting information can be downloaded at: https://www.mdpi.com/article/10.3390/biom16040534/s1, Figure S1: Volcano plot active versus inactive uveitis; Figure S2: Volcano plot with glaucoma versus without glaucoma; Table S1: Clinical data; Table S2: DEGs group comparisons; Table S3: DEGs transcripts of ANA-U and JIAU/ANA-U during active uveitis shown in a VENN diagramm in Figure 4C; Table S4: Gene ontology; Table S5: DEGs transcripts of JIAU, ANA-U and JIAU/ANA-U with glaucoma shown in a VENN diagramm in Figure 6C.

## Abbreviations

ANAs: anti-nuclear antibodies, AU: anterior uveitis, AAU: acute anterior uveitis, cDNA: complementary desoxyribonucleic acid, DEGs: differentially expressed genes, DMARD: disease-modifying anti-rheumatic drug, FDR: false discovery rate, HLA: human leukocyte antigen, ILAR: International League of Associations for Rheumatology, IOP: intraocular pressure, JIA: juvenile idiopathic arthritis, JIAU: juvenile idiopathic arthritis-associated uveitis, m: male, mRNA: messenger RNA, MTX: methotrexate, PCA: principle component analysis, RF: rheumatoid factor, RNA: ribonucleic acid, RNA-Seq: RNA Sequencing, SUN: standardization of uveitis nomenclature.
